# Transcriptional Analysis of the Conjugal Transfer Genes of *Rickettsia bellii* RML 369-C

**DOI:** 10.1371/journal.pone.0137214

**Published:** 2015-09-09

**Authors:** Chan C. Heu, Timothy J. Kurtti, Curtis M. Nelson, Ulrike G. Munderloh

**Affiliations:** Department of Entomology, University of Minnesota, 219 Hodson Hall, 1980 Folwell Ave, St. Paul, Minnesota, United States of America; Washington State University, UNITED STATES

## Abstract

*Rickettsia bellii* is an obligate intracellular bacterium that is one of the few rickettsiae that encode a complete set of conjugative transfer (*tra*) genes involved in bacterial conjugation and has been shown to exhibit pili-like structures. The reductive genomes of rickettsiae beg the question whether the *tra* genes are nonfunctional or functioning to enhance the genetic plasticity and biology of rickettsiae. We characterized the transcriptional dynamics of *R*. *bellii tra* genes in comparison to genes transcribed stably and above the background level to understand when and at what levels the *tra* genes are active or whether the *tra* genes are degenerative. We determined that the best reference genes, out of 10 tested, were methionyl tRNA ligase (*metG*) or a combination of *metG* and ribonucleoside diphosphate reductase 2 subunit beta (*nrdF*), using statistical algorithms from two different programs: Normfinder and BestKeeper. To validate the use of *metG* with other rickettsial genes exhibiting variable transcriptional patterns we examined its use with *sca2* and *rickA*, genes involved in actin based motility. Both were shown to be up-regulated at different times of replication in Vero cells, showing variable and stable transcription levels of *rickA* and *sca2*, respectively. *traA*_*Ti*_ was up-regulated at 72 hours post inoculation in the tick cell line ISE6, but showed no apparent changes in the monkey cell line Vero and mouse cell line L929. The transcription of *tra* genes was positively correlated with one another and up-regulated from 12 to 72 hours post inoculation (HPI) when compared to RBE_0422 (an inactivated transposase-derivative found within the *tra* cluster). Thus, the up-regulation of the *tra* genes indicated that the integrity and activity of each gene were intact and may facilitate the search for the optimal conditions necessary to demonstrate conjugation in rickettsiae.

## Introduction

Rickettsiae are obligate-intracellular, coccobacillary, Gram-negative bacteria that include invertebrate symbionts. Genetic analysis of rickettsiae has revealed that plasmids are widely distributed within the genus [1,2] and *tra* genes are common [3], suggesting that rickettsiae can transfer mobile genetic elements, i.e. plasmids, to receptive bacteria via conjugation. *Rickettsia bellii* strain RML 369-C [4], *Rickettsia massiliae* MTU5 [5], *Rickettsia felis* strain LSU-lb [6], and the rickettsial endosymbiont of *Ixodes scapularis* (REIS, i.e. *Rickettsia buchneri*) [7,8] possess a set of *tra* genes that encodes an apparently complete bacterial conjugation system, but it is not known whether they are functional. In contrast, several other rickettsiae, e.g. *Rickettsia felis* strain California 2, only encode a few of the *tra* genes, suggestive of an incomplete conjugation system [2]. Nevertheless, pili-like structures have been observed in *R*. *felis* as well as *R*. *bellii* [2,4,9]. Furthermore, the *tra* clusters of *R*. *bellii* RML 369-C, *R*. *felis* LSU-lb, and *R*. *massiliae* MTU are similar at the nucleotide sequence level, gene order, and gene orientation to at least one of the *tra* clusters of *R*. *buchneri*, suggesting multiple lateral gene transfer events between rickettsiae [7,10]. Despite this, bacterial conjugation has not been documented in any rickettsiae.

**Fig 1:**
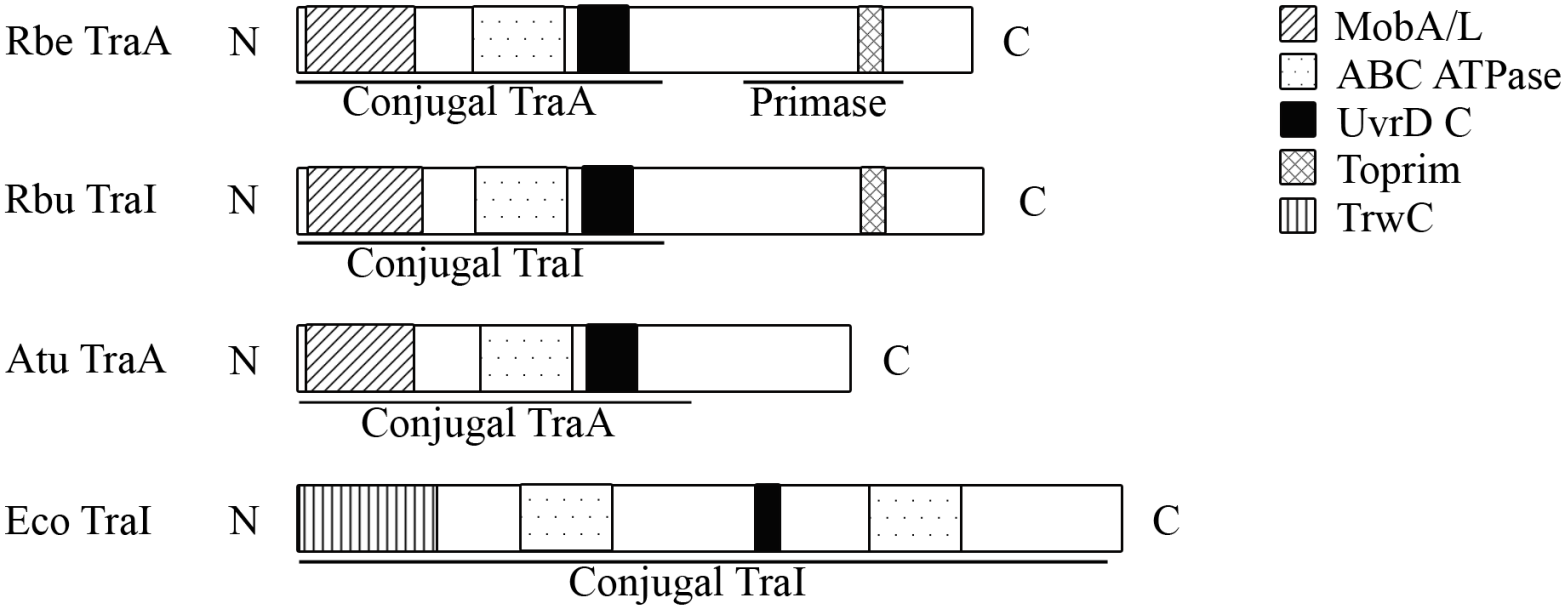
Graphical display of the domains of *R*. *bellii* TraA_Ti_ and its related protein. MobA/L: this domain is responsible for specific DNA strand transfer in the donor bacterium; ABC ATPase: functions in ATP and GTP binding and hydrolysis, which is a characteristic of conjugative proteins; UvrD C: this domain is found at the carboxyl terminal end of helicases suggesting their role in helicase activity; Toprim: a domain found in DnaG-type primases; TrwC: this domain signifies the relaxase in conjugative DNA transfer during cell-to-cell contact; Conjugal TraA/I: a protein segment involved in DNA binding, nicking, and helicase activity; Primase: a protein segment involved in priming of DNA. The function of each domain was assigned by the Conserved Domain Database. Rbe = *R*. *bellii*, Rbu = *R*. *buchneri*, Atu = *A*. *tumefaciens*, Eco = *E*. *coli*, N = amino terminal end of the protein, and C = carboxyl terminal end of the protein.

The initiation of conjugation in *Escherichia coli*, the model organism, begins with pilus contact with a recipient cell followed by retraction of the pilus and the formation of a stable mating pair [11]. The timing and signal to process the transfer DNA is unknown; nevertheless, the mechanism of generating the transfer DNA has been established. Briefly, TraI is the main processing enzyme with nickase and helicase activity [12,13] that covalently binds to the transfer DNA with the help of accessory proteins [14–16]. Subsequently, TraI-bound DNA docks with TraD [17], an inner membrane coupling protein that initiates the helicase activity of TraI to generate a single-stranded transfer DNA. TraD then opens the pore for transport of the nucleoprotein complex to the recipient thus completing conjugation. *E*. *coli* has been shown to express TraI until late stationary phase; however, mating success was highest from mid-log phase to early stationary phase [18]. TraI shows homology to TraA_Ti_ in *R*. *bellii* at the protein level, sharing similar domains with presumably similar functions (Fig 1) as well as belonging to the same class of enzyme, relaxases [19]. As seen in *E*. *coli* [18], certain growth phases of rickettsiae may correlate with transcription of *traA*_*Ti*_ and other *tra* genes, suggesting that conjugation occurs around the time of highest transcription.

The aim of this research was to follow the transcriptional patterns of *traA*_*Ti*_ in *R*. *bellii* in comparison with stably transcribed reference gene(s) to facilitate the assessment of the function of the *tra* genes. We determined the best reference gene at different times of growth in ISE6 (*I*. *scapularis* tick embryonic cells), Vero (African green monkey kidney cells) and L929 (mouse fibroblast cells), all of which support continuous *R*. *bellii* replication, and may be taken to represent the rickettsial life cycle alternating between the arthropod and the mammalian host. A two-step quantitative reverse transcription PCR (qRT-PCR) and two specialized statistical programs, Normfinder [20] and BestKeeper [21], were used to screen 10 housekeeping genes in *R*. *bellii*: *16S rRNA*, *atpB*, *dnaK*, *gltA*, *gyrA*, *infB*, *metG*, *nrdF*, *rpoB*, and *tlc5* (S1 Table). Of these, *metG* was found to be the most stably transcribed throughout the infection cycles. We validated this as our reference gene by using it to demonstrate the differential transcription patterns of *rickA* and *sca2* as reported [22]. We then used *metG* to analyze the transcription of the components of *R*. *bellii tra* cluster, *traA*_*Ti*_, *traD*_*Ti*_, *traL*, *traE*, *traB*, *traV*, *traC*, *traW*, *traU*, *trbC*, *traN*, *traF*, *traH*, *traG*, *traD*_*F*_, and RBE_0422 during growth in ISE6 cells, and we used *metG*, *nrdF*, and a combination of *metG* and *nrdF* for a comparative analysis of transcription activity of *traA*_*Ti*_ during replication in different host cells in vitro.

## Materials and Methods

### Determining the domains of TraA_Ti_ and related proteins

The location and functions of the domains of TraA_Ti_ of *R*. *bellii* RML 369-C (YP_537591.1), TraI of *R*. *buchneri* (KDO03561.1), TraA of *Agrobacterium tumefaciens* (WP_035228570.1), and TraI of *E*. *coli* (WP_038999217.1) were identified using Batch CDD search [23] on the Conserved Domain Database site (http://www.ncbi.nlm.nih.gov/Structure/cdd/cdd.shtml). The graphical representations of the domains of each protein were drawn in Adobe Photoshop to illustrate the location and overall function of each segment of the proteins (Fig 1).

### Host cells

Cell line ISE6 [24] was cultured at 34°C using a modified Leibovitz’s L15 medium (L15C-300) supplemented with 5% fetal bovine serum (FBS), 5% tryptose phosphate broth, and 0.1% lipoprotein concentrate as described [25,26]. Vero (ATCC CCL-81) and L929 (ATCC CCL-1) were cultured at 37°C using RPMI+GlutaMAX 1640 medium (Gibco, Grand Island, NY) supplemented with 10% FBS and 2 mM L-glutamine.

### Preparation of cell-free *R*. *bellii* stabilate for inoculation of host cell cultures

*R*. *bellii* RML 369-C was grown in ISE6, Vero and L929 cells in 25 cm^2^ tissue culture flasks (BD Falcon, NJ) at 34°C in L15-C300 supplemented with 10% FBS for at least 2 subcultures within the respective host cells before preparing and freezing cell-free rickettsia stocks. In brief, infected cells were scraped off the flask, suspended in L15C-300 medium, transferred to 1.5 ml microcentrifuge tubes containing 60–90 grit silicone carbide (Lortone, Inc., Mukilteo, WA), and vortexed at maximum speed for at least 20 seconds to rupture host cells and obtain cell-free *R*. *bellii*. Liberated *R*. *bellii* were filter-purified using a Whatman 2.0 μm filter (GE Healthcare Life Science, NJ) as described in Oliver *et al*. (2014), collected by centrifugation at 13,000x rcf for 5 minutes at 4°C and resuspended in freezing medium (L15C-300 with 50% FBS and 10% DMSO), then aliquoted into cryogenic vials and frozen at a controlled rate of -1°C/min for storage in liquid nitrogen.

### Inoculation of host cell cultures

Uninfected cells were seeded into 12.5 cm^2^ tissue culture flasks (BD Falcon, NJ) with 3 flasks for each time point and cells were allowed to attach overnight. Before inoculation, the multiplicity of infection was determined by counting rickettsiae and host cells. Rickettsiae were visualized using LIVE/DEAD BacLight Viability Kit (Molecular Probes, OR) and quantified using a Petroff-Hauser counting chamber. The number of host cells in triplicate cultures was quantified using an Improved Neubauer counting chamber. The number of live rickettsia divided by the host cell counts yielded the multiplicity of infection. Subsequently, the rickettsial stabilates were diluted to the point where inoculating host cell layers with a total volume of 200 μl resulted in 10–50 rickettsiae per host cell. Immediately following inoculation, cultures were transferred to 4°C for 1 hour to allow binding of rickettsiae to cells and to synchronize the infection. Cell cultures were shifted from 4°C to 34°C and cultures incubated at 34°C for 1 hour to facilitate endocytosis of bound rickettsiae. Cell layers were washed twice with 1x phosphate buffered saline containing calcium and magnesium to remove unbound rickettsiae. Fresh L15C-300 with 10% FBS was added to infected cell layers and cultures incubated at 34°C for 12, 24, 36, 48, and 72 HPI for infected ISE6 cultures and 12, 24, 36, 48, 60, 72, 84, and 96 HPI for infected Vero and L929 cultures.

### Quantification of *R*. *bellii*

Quantification methods were validated by comparing purified cell-free *R*. *bellii* (rickettsiae prepared, RP) with yields from host cells infected with *R*. *bellii* (whole cell, WC) at 12 and 72 HPI. The total number of *R*. *bellii* obtained from each extraction methods was determined by qPCR, using a standard curve based on quantification of a single copy gene, *gltA*. Statistical significance was measured using a two-sided two sample Student’s t-test with significance set at an alpha of 0.05.

Rickettsial DNA and RNA were extracted from each culture. Whole cell lysis of rickettsiae and host cells was initially used for preparing total DNA and RNA. However, first strand cDNA synthesis using random hexamer yielded low rickettsial cDNA at earlier times post inoculation of rickettsiae; thus, cell-free rickettsial preparation were used. Cell cultures were harvested and RP were prepared with silicone grit as stated previously and stored at -80°C. Pellets were thawed and immediately lysed for DNA and RNA extraction using ZR-Duet DNA/RNA MiniPrep kit (Zymo Research, CA) according to the manufacturer’s protocols. Extracted DNA and RNA were stored at -20°C and -80°C, respectively. qPCR was used to quantify the *gltA* copy numbers from purified DNA of liberated *R*. *bellii* grown in ISE6, Vero, and L929 [1,27]. The pCR4 TOPO (Invitrogen, NY) plasmid fused with the PCR product of Rbellii qCSF/R primers (S1 Table) and the respective primers were used to generate the standard curve for quantifying absolute copy numbers of *R*. *bellii* for each time point using Brilliant II SYBR Green qPCR Low Rox Master Mix (Agilent, CA) at 1x with a final concentration of 0.08 μM of each primer, and 5 μl of the total eluted *R*. *bellii* DNA. The cycling parameters were 95°C for 10 minutes, followed by 40 cycles of 95°C for 30 seconds, 55°C for 1 minute, and 72°C for 30 seconds. A dissociation curve was used to confirm a single PCR product with a cycle starting at 95°C for 1 minute, 55°C for 30 seconds, and 95°C for 30 seconds immediately after the quantification cycles.

### Growth of *R*. *bellii* in tick and mammalian cell cultures

Cell cultures infected with *R*. *bellii* were extracted for DNA at 12, 24, 36, 48, and 72 HPI for ISE6 and additional times of 60, 84, and 96 HPI for Vero and L929. Rickettsiae numbers were quantified as previously mentioned. The values from triplicate cultures at each time point were averaged and graphed using SigmaPlot 13 (Systat Software, Inc., CA).

### Selection, quantification, and validation of *R*. *bellii* reference gene transcripts

To identify stable reference genes to analyze the transcriptional responses of the *tra* genes in *R*. *bellii* grown for different times (12, 24, 36, 48, and 72 HPI) and in different host cells (48 HPI only), a two-step qRT-PCR was used employing sample maximization [28]. Reference genes (*16S rRNA*, *atpB*, *dnaK*, *gltA*, *gyrA*, *infB*, *metG*, *nrdF*, *rpoB*, and *tlc5*) were selected based on their predicted molecular function in different metabolic classes and their property as housekeeping genes (S1 Table). In addition, five out of the ten reference genes were selected to show transcription levels above background in *Rickettsia rickettsii* Sheila Smith based on tiling array data (S1 Text, S1 Fig, S2 Table).

First strand cDNA was synthesized from total RNA using random hexamer primers (Integrated DNA Technology, IA) and Superscript II Reverse Transcriptase (Life Technologies, NY) following the manufacturer’s protocol and stored at -20°C. All cDNA was diluted 1:20 with water and 5 μl of the dilution was added to each qPCR reaction. Master mix and cycling parameters were similar to the conditions used to determine *gltA* copy numbers, except the primer hybridization temperature for *traV* detection was lowered to 50°C based on the GC content of the primer set used. The transcript numbers were normalized using the ratio of *gltA* genomic copy numbers detected at all time points to 12 HPI.

Normfinder and BestKeeper (http://moma.dk/normfinder-software and http://www.gene-quantification.com/bestkeeper.html, respectively) were used to rank and identify the best reference gene or combination of reference genes to use for comparative analysis. Normfinder uses a model based approach to determine the most stable gene [20] and can handle high variations from low copy numbers of transcript from earlier time points because of lower number of rickettsiae and high copy numbers at later times. The ranking of our genes using BestKeeper was based on the highest coefficient of correlation for the best reference gene to the lowest coefficient of correlation for the least favorable reference gene [21]. BPROM, a promoter prediction program (http://linux1.softberry.com/berry.phtml?topic=bprom&group=programs&subgroup=gfindb), was used to identify potentially shared promoters upstream of the best reference genes identified by BestKeeper to discount similarly regulated genes for use as reference genes.

### Transcription of *rickA* and *sca2* in Vero cells to validate *metG* as a reference gene

Validation of *metG* as the best reference gene was accomplished using primers to determine the transcription pattern of *rickA* and *sca2*. The differences within and between the transcription patterns of *rickA* and *sca2* were statistically analyzed using multiple comparisons with an ANOVA test and a Holm-Sidak correction with an alpha value set at 0.05 in SigmaPlot 13.

### Relative transcription of *traA*_*Ti*_ to reference genes

First strand cDNA synthesis was generated, as mentioned previously, from RNA of *R*. *bellii* isolated from infected ISE6, Vero, and L929 cells at all the time points. Two-step qRT-PCR was done using primers for *traA*_*Ti*_ and for the reference genes *metG* and a combination of *metG* and *nrdF*. Template consisted of 1^st^ strand cDNA synthesized at all time points in all cell lines. The 2^−ΔΔCT^ method was used to quantify fold changes of *traA*_*Ti*_:*metG* at all time points relative to 12 HPI [29]. SigmaPlot 13 was used to generate line graphs.

### Transcriptional analysis of the *tra* from 12 to 72 HPI

Transcriptional analysis of *traA*_*Ti*_, *traD*_*Ti*_, *traL*, *traE*, *traB*, *traV*, *traC*, *traW*, *trbC*, *traU*, *traF*, *traH*, *traG*, *traD*_*F*_, and RBE_0422 was carried out under similar conditions and cycling parameters, as previously stated, using cDNA from 12 and 72 HPI of *R*. *bellii* grown in ISE6 as templates together with respective primer sets (S1 Table).

## Results

### Domains of *R*. *bellii* TraA_Ti_ are related to conjugative relaxases of other bacterial species

The domains of *R*. *bellii* TraA_Ti_ are depicted in Fig 1. The MobA/L, ABC ATPase, and UvrD C domains of *R*. *bellii* TraA_Ti_ and *R*. *buchneri* TraI constitute the conjugal segment of TraA. This conjugal segment is predicted to have similar function as the conjugal segment of *A*. *tumefaciens* TraA and *E*. *coli* TraI (underlined in Fig 1) such as DNA binding, nicking, and unwinding [12,13,30] when generating an ssDNA for DNA transfer, which are all qualities of relaxases although the amino acid sequences may vary between the families of relaxases [31] In addition to the conjugal segment of TraA_Ti_ of *R*. *bellii*, a primase was identified with a Toprim domain at the carboxyl terminal end. The overall domains of *R*. *bellii* TraA_Ti_ are similar to the well characterized conjugal relaxases of other bacteria; thus, we suspect that *R*. *bellii* TraA_Ti_ expression may be a good indicator of the time of induction for potential bacterial conjugation.

### Growth of *R*. *bellii* in tick and mammalian cell cultures

To verify that there were no differences in rickettsial numbers determined from RP vs. WC, *R*. *bellii* DNA was prepared and the *gltA* genomic copy numbers in each preparation were compared (Table 1). According to the two-sided two sample Student’s t-test with alpha set to 0.05, no significant differences were observed between the two isolation procedures. Thus, the process of preparing cell-free rickettsial preparations did not cause a significant loss of rickettsiae when compared with whole cell lysate.

**Table 1:** A comparison of *gltA* copy numbers of RP and WC *R*. *bellii*.

Time	12 HPI		72 HPI	
Method	RP	WC	RP	WC
Mean	4.01x10^7^	3.77x10^7^	3.72x10^9^	4.13x10^9^
S.E.	6.98x10^6^	1.05x10^7^	2.04x10^8^	2.62x10^8^
p ≤0.05	0.86		0.28	

A two-sided two sample Student’s t-test with an alpha value at 0.05 was used to compare the number of detected *gltA* copy numbers in rickettsiae prepared (RP) by semi-purification and whole cell (WC) lysis of *R*. *bellii* RML 369-C grown in ISE6 at 34°C. Results show no significant difference between the two methods.

**Fig 2:**
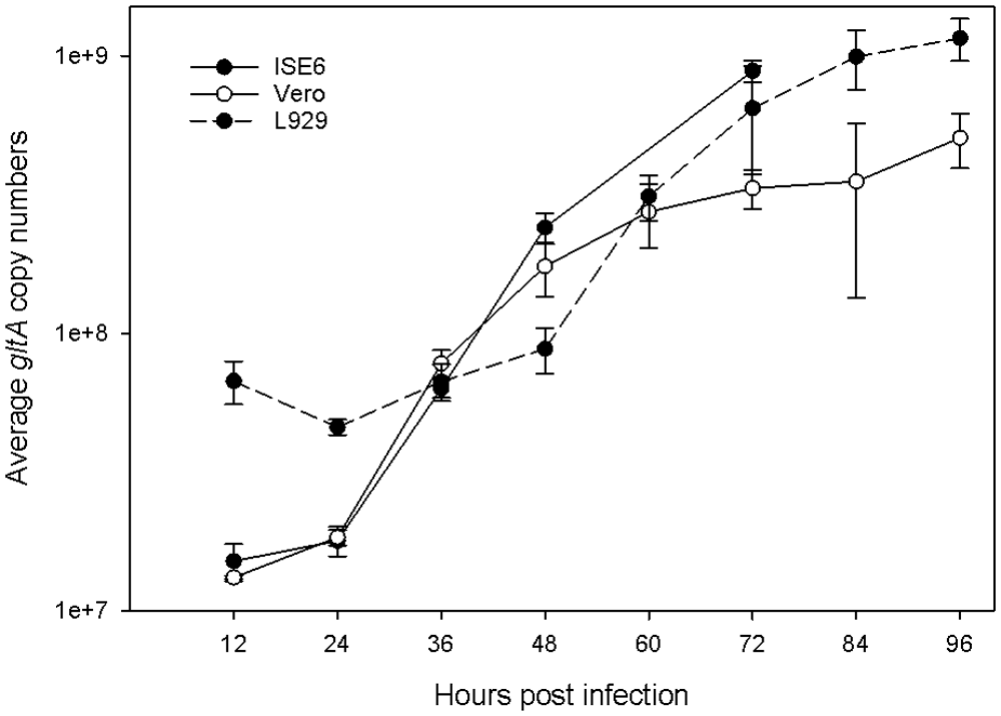
Growth curve of *R*. *bellii* in ISE6, Vero, and L929. qPCR detected a single copy gene of *gltA* for triplicates of culture at each time point. Standard error bars are shown. Growth trials using Vero and L929 were done simultaneously while ISE6 was done separately.

Subsequently, *gltA* copy numbers were quantified using the RP method and qPCR for all time points and host cells. The growth curves for *R*. *bellii* in different cell lines (Fig 2) shared similar growth phases and a doubling time of approximately 8 hours during the period of 36 to 60 HPI similar to times reported for *Rickettsia prowazekii* and *Rickettsia rickettsii* [32–34]. The initial higher copy numbers of *gltA* from *R*. *bellii* isolated from L929 could have resulted from inefficient washing or highly efficient phagocytosis and killing of rickettsiae, but slower digestion of rickettsial DNA by L929. We concluded that the replication of *R*. *bellii* was similar in the different host cells using an MOI of 10–50 followed by a wash step.

### Identifying the best reference gene and best combination of genes

Ten *R*. *bellii* housekeeping genes were selected based on their different functional classes, i.e. translation, DNA replication: *16S rRNA*, *atpB*, *dnaA*, *gltA*, *gyrA*, *infB*, *metG*, *nrdF*, *rpoB*, and *tlc5* (S1 Table). Results for *R*. *rickettsii* microarray analysis showed transcription levels above background for each of the selected genes (S1 Fig). Normfinder and BestKeeper were used to analyze the transcript data of the *R*. *bellii* reference genes (Table 2). Normfinder ranked *metG* as the most stably transcribed gene throughout a 72 HPI growth period in ISE6 and at 48 HPI between the different host cell lines. Normfinder identified a combination of *metG* and *nrdF* for transcriptional analysis throughout a 72 HPI in ISE6, while *gyrA* and *metG* were selected by Normfinder when comparing between host cells at 48 HPI. In comparison, BestKeeper ranked *gyrA* and *metG* as the top two genes to use for a time point analysis and for comparing between host cells based on the coefficient of correlation (Table 2). A caveat of BestKeeper is that it can identify reference genes that are transcriptionally co-regulated, which is not ideal when identifying the best reference gene. To avoid co-regulated transcription, the selected genes were chosen based on their different molecular functions that belong to different metabolic classes, i.e. *gyrA* and *metG* functions in DNA replication and initiation of translation, respectively, suggesting that the genes tested are not co-regulated. Analysis of the genetic sequences upstream of *metG* and *gyrA* using BPROM identified a promoter upstream of *gyrA* at position 879453 with a linear discriminant function score of 9.77, and located a -10 box at 879468 with a score of 61, and a -35 box at 879492. Also, *metG* has a promoter at position 282683 with linear discriminant function score of 9.18, a -10 box at 282668 with score of 63, and a -35 box at 282649 with a score of 47. Both promoters were shown to have several transcription factor binding sites, but share only a leucine-responsive regulatory protein binding site. This transcription factor is involved in controlling the cell response to nutritional stress, particularly to leucine levels [35]. However, *R*. *bellii* does not encode a leucine-responsive regulatory protein-like protein according to the annotated *R*. *bellii* RML 369-C genome, indicating that *metG* and *gyrA* are not similarly regulated. Overall, *metG* is the best reference gene selected by Normfinder and BestKeeper either as the best or second best gene to use as a reference when comparing rickettsiae replicating in different host cells.

**Table 2:** The rankings of each candidate gene by Normfinder and BestKeeper.

	Normfinder		BestKeeper	
	Stability Value		Coefficient of Correlation	
Gene name	Different HPI in ISE6	Different host cells	Different HPI in ISE6	Different host cells
*16S rRNA*	0.233	0.471	0.916	0.872
*atpB*	0.216	0.314	0.923	0.918
*dnaK*	0.129	0.116	0.983	0.973
*gltA*	0.314	0.373	0.993	0.978
*gyrA*	0.157	0.093*	0.995**	0.987**
*infB*	0.212	0.227	0.965	0.960
*metG*	0.080**	0.080**	0.994*	0.983*
*nrdF*	0.117*	0.211	0.990	0.952
*rpoB*	0.232	0.373	0.920	0.904
*tlc5*	0.315	0.147	0.921	0.981

** = most stable reference gene,

* = second most stable reference gene.

### Transcription of *rickA* and *sca2* is induced at different growth phases in Vero cells, initially validating *metG* as a reference gene

**Fig 3:**
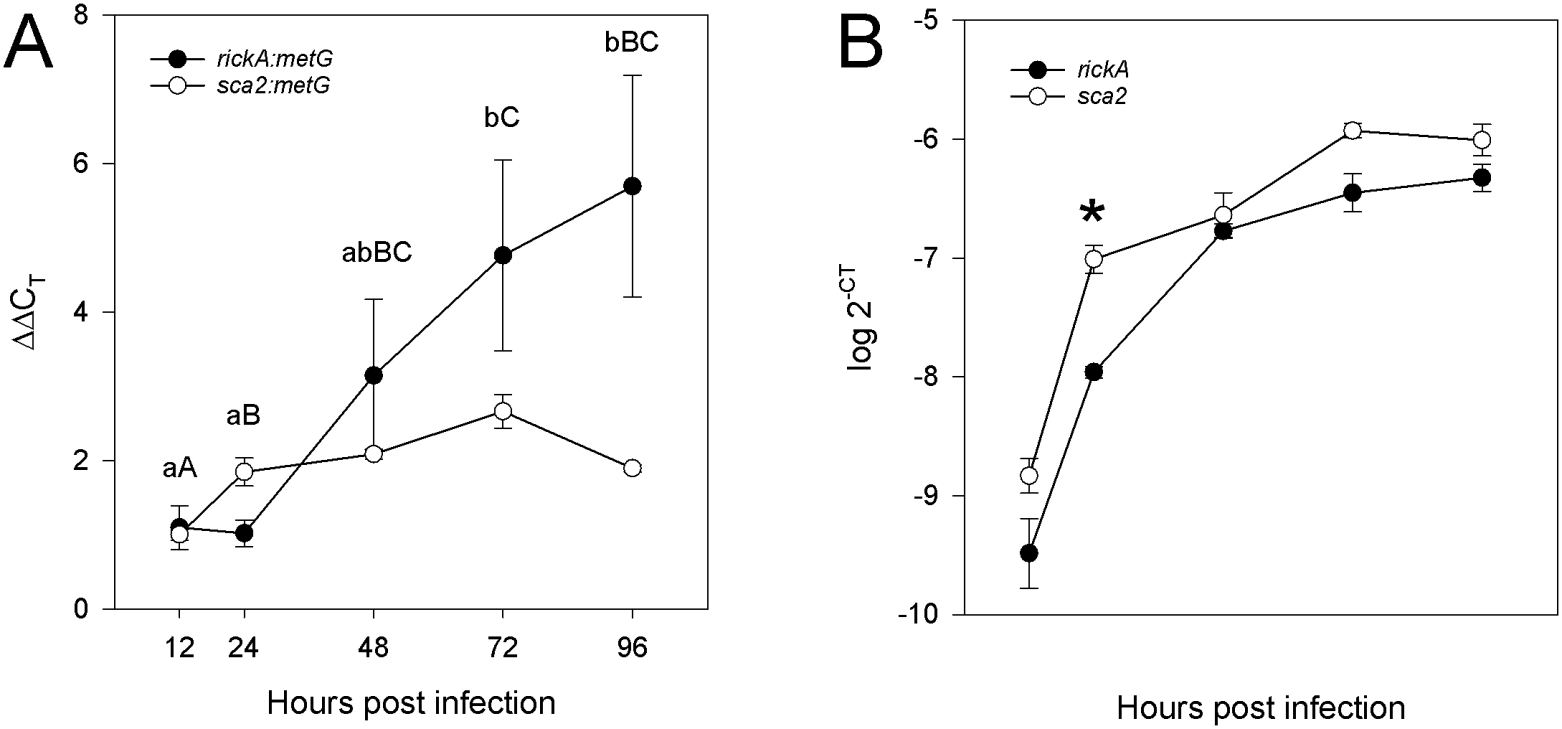
Initial validation of reference genes. *rickA* and *sca2* of *R*. *bellii* grown in Vero cells were selected to demonstrate differential gene transcription using *metG* as a reference. (**A**) The line graph depicts the fold changes of *rickA* and *sca2* relative to *metG* at all time points to 12 HPI. Letters indicate significant difference within each gene at different times (lower case = *rickA*, upper case = *sca2*) (**B**) The graph shows the transcription level of *rickA* and *sca2* at each time point and (*) indicates significant difference. An ANOVA with Holm-Sidak correction was used to determine significant differences with alpha set at 0.05. Standard error bars are shown.

*R*. *bellii* uses actin-based motility similar to other intracellular bacteria [25]. Thus far, *rickA* and *sca2* were found to be involved in actin tail polymerization [36,37], and their products, RickA and Sca2, were shown to be expressed during distinct phases of intracellular growth [22]. Therefore, we were interested in defining the transcriptional patterns of *rickA* and *sca2* to validate our reference gene. Relative transcription of *rickA* and *sca2* was determined as described using *metG* as the reference gene. Up-regulation of *sca2* was observed from 12 to 24 HPI (Fig 3A), which correlated with the lag phase of the growth curve (Fig 2). *rickA* showed a trend of up-regulation between 48 to 96 HPI with significant differences in transcription levels at 72 and 96 HPI that coincided with the mid-log phase to the stationary phase. *sca2* showed a significant difference in relative transcription level between 12 and 24 HPI that coincided with the lag phase and between 24 and 72 HPI that coincided with the beginning of the log phase and stationary phase, respectively (Fig 3A). The transcription patterns of *sca2* and *rickA* were modulated by distinct phases of infection and growth. Analyses of *rickA* and *sca2* transcription showed significant differences between each other at 24 HPI (Fig 3B). Furthermore, *rickA* and *sca2* transcript levels displayed a trend similar to the growth curve (Figs 1 and 3B). Thus, *sca2* may play an important role during the lag phase of invasion especially during the beginning of the log phase, while *rickA* may have an important role in the exponential phase of *R*. *bellii* replication.

### Transcription of *traA*_*Ti*_ in *R*. *bellii* is induced in ISE6 at 72 HPI

**Fig 4:**
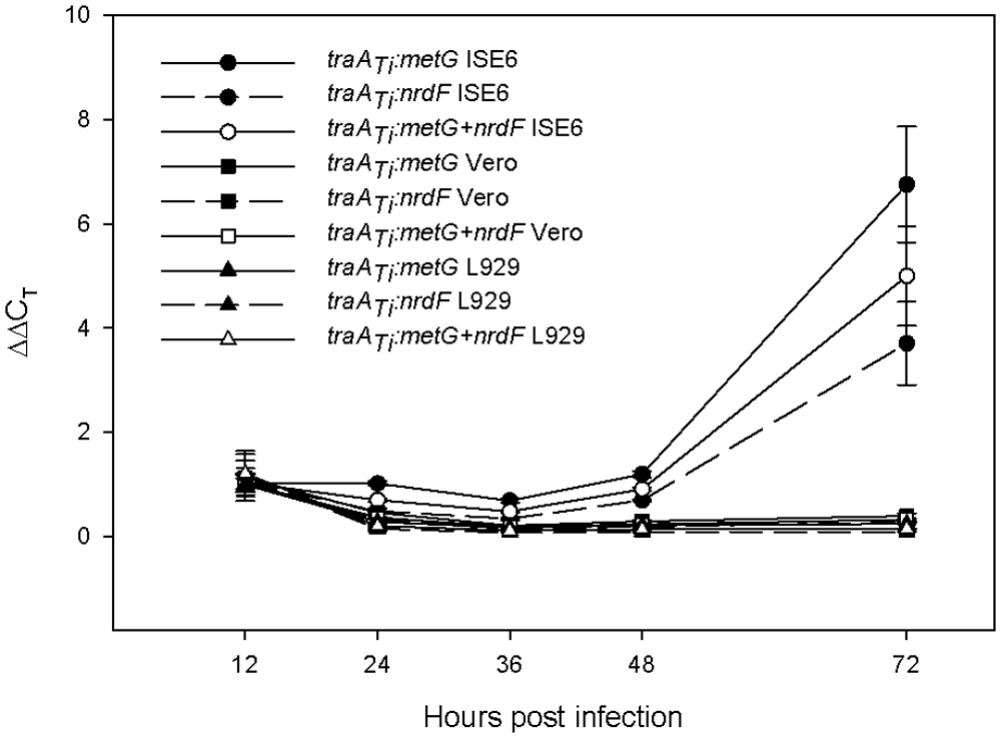
Fold change of *traA*_*Ti*_ in different host cells at different hours post infection. qRT-PCR was used to determine the *traA*_*Ti*_ transcription pattern using *metG* only, *nrdF* only, and a combination of *metG* and *nrdF*. Standard error bars are shown.

Two-step qRT-PCR was used to quantify *traA*_*Ti*_ mRNA through time in different host cells using *metG* and a combination of *metG* and *nrdF* as reference genes. At 24, 36, and 48 HPI *traA*_*Ti*_ in *R*. *bellii* grown in ISE6 did not show any apparent differences in transcription when compared to 12 HPI using *metG* only, *nrdF* only, or *metG* and *nrdF* as reference genes, but at 72 HPI *traA*_*Ti*_ was up-regulated compared to 12 HPI (Fig 4). In addition, *traA*_*Ti*_ was also up-regulated in *R*. *bellii* grown in ISE6 in comparison to *R*. *bellii* grown in Vero and L929 at 72 HPI (Fig 4). As a result of the induction of *traA*_*Ti*_ observed at 72 HPI, *R*. *bellii* was grown in ISE6 for 12 and 72 HPI and examined for the transcriptional patterns of the other annotated *tra* genes. All examined *tra* genes were found to be up-regulated at 72 HPI (Table 3) as indicated by the lower C_T_ values where the threshold is reached with lower number of cycles when starting with a greater quantity of template.

**Table 3:** C_T_ values of *tra* genes and *metG* at 12 and 72 HPI.

*tra* genes	*metG* 12 HPI	*metG* 72 HPI	*tra* 12 HPI	*tra* 72 HPI	ddC_T_
*traL*	27.65±0.05	25.44±0.23	33.49±0.16	28.91±0.25	**5.12±0.08**
*traE*	27.65±0.05	25.44±0.23	36.76±0.35	31.98±0.38	**6.01±0.64**
*traB*	25.85±0.06	23.7±0.27	33.64±0.26	28.42±0.18	**8.45±0.53**
*traV*	27.01±0.06	24.98±0.21	36.38±0.13	31.19±0.26	**8.94±0.46**
*traC*	25.85±0.06	23.7±0.27	35.63±0.09	30.27±0.21	**9.39±0.68**
*traW*	25.85±0.06	23.7±0.27	30.76±0.12	25.92±0.21	**6.48±0.27**
*traU*	27.65±0.05	25.44±0.23	36.92±0.02	31.21±0.34	**11.35±0.83**
*trbC*	27.65±0.05	25.44±0.23	35.76±0.12	30.01±0.23	**11.20±0.47**
*traN*	27.19±0.05	24.9±0.25	33.75±0.24	28.9±0.29	**5.92±0.17**
*traF*	27.19±0.05	24.9±0.25	ND	35.49±0.20	**NA**
*traH*	27.19±0.05	24.9±0.25	34.2±0.11	29.39±0.22	**5.80±0.35**
*traG*	27.19±0.05	24.9±0.25	32.91±0.05	26.37±0.20	**19.20±0.91**
*traD*_*F*_	26.56±0.02	24.2±0.31	26.69±0.03	23.26±0.25	**2.11±0.08**
*traA*_*Ti*_	26.56±0.02	24.2±0.31	31.81±0.13	27.55±0.23	**3.75±0.21**
*traD*_*Ti*_	25.32±0.08	23.11±0.15	36.53±0.15	31.56±0.22	**6.81±0.38**
*Rbe_0422*	30.47±0.14	24.36±.27	31.63±0.08	25.20±0.36	**1.25±0.08**

C_T_ values are given with standard errors for *metG* and *tra* genes at 12 and 72 HPI. ND = No detection, NA = Not available.

## Discussion

Several studies have analyzed relative transcription patterns in rickettsiae, e.g. using *16S rDNA*, *metG*, and *gltA* [25,38,39]. However, the reference genes chosen were not rigorously evaluated and validated as the most stably transcribed genes under experimental conditions using statistical programs. In this study, we evaluated 10 different genes and identified and validated *metG* or *metG* and *nrdF* as the best reference genes to use for transcriptional analysis of the *tra* genes during *R*. *bellii* growth in ISE6 cell cultures and in different mammalian cell lines using NormFinder. BestKeeper identified *gyrA* and *metG* as the top two reference genes. Based on the promoters (predicted by BPROM) and the differences in molecular functions between *metG* and *gyrA*, we surmised that they were unlikely to be transcriptionally co-regulated. *metG* and *nrdF* were also shown to be constitutively transcribed in *R*. *bellii* (S2 Fig). Tiling array data from *R*. *rickettsii* also showed that transcription levels of *metG*, *nrdF*, *gyrA*, *gltA*, and *16S rDNA* were above background (S1 Fig). Both results corroborated the stability of the transcription of *metG*, *nrdF*, and *gyrA* indicating that all three may be good reference genes. However, *metG* consistently ranked as the best reference gene to be used for relative transcriptional analysis. *metG* was also used to determine the transcriptional response of *R*. *rickettsii* to a shift in temperature suggesting that both pathogenic and nonpathogenic rickettsiae may transcribe *metG* at stable levels under different treatment conditions.

We further validated our reference genes by tracking transcriptional patterns of *sca2* and *rickA*. We observed a differential transcription pattern of *sca2* and *rickA*, with a distinctive up-regulation of *sca2* during the lag phase of rickettsial growth and a trend of increasing variance of the transcription of *rickA* during exponential phase. Sca2 up-regulation at 24 HPI suggested that it played an important role when *R*. *bellii* was invading and adapting to the host cells until it was ready for the exponential phase (Fig 3A). In contrast, *rickA* demonstrated high variability of transcription levels during the mid-exponential growth phase to the stationary phase suggesting discontinuous and variable motility of *R*. *bellii* (Fig 3A). This variability of *rickA* transcript may be attributed to the first asynchronous infection of neighboring cells near 48 HPI when rickettsiae begin to spread and infect new cells [33,40]. Both *rickA* and *sca2* had general transcription patterns that trended with the growth curve of *R*. *bellii* except at the beginning of the exponential phase (24 HPI) where *sca2* transcription was significantly different from *rickA* (Fig 3B). Sca2 is found at the surface [41] and has been shown to be involved in adhesion to mammalian cells [42]. Thus, *sca2* is probably transcribed significantly more than *rickA* at the initial part of the exponential phase to express enough Sca2 to ensure that they can continue to adhere to and invade host cells, as well as to enable them to use actin-base motility for later stages of cell-to-cell spread as indicated by Reed *et al*. 2014 [22]. Expression patterns of RickA and Sca2 in *Rickettsia parkeri* were significantly different at early and late infections correlating with actin-tail morphology and were observed along with *rickA* or *sca2* knockouts to determine the differences in cell-to-cell spread [21]. In contrast, our results indicated that *rickA* and *sca2* were differentially transcribed at different times post infection [22] to highlighting the importance of *rickA* and *sca2* at different growth phases of *R*. *bellii*. Furthermore, Sca2 of *R*. *bellii* lacks the formin homology 2 domain, but maintains the formin homology 1 domain, and has been shown to sufficiently accelerate actin polymerization indicating it functions as a tandem monomer-binding nucleator [43]. These differences may result in different regulation of expression and function of *sca2* and indirectly affecting *rickA*. Nonetheless, we demonstrated that a significant difference in the transcription levels of *rickA* and *sca2* of *R*. *bellii* existed during the transition of lag to exponential growth phase. Thus, this is the initial validation of *metG* as a suitable reference gene.

TraA_Ti_ was chosen as the indicator of the conditions to examine transcription of the other *tra* genes because it was shown to reflect responses of the entire *tra* cluster. TraA_Ti_ has domains that suggest it is functionally similar to TraI (Fig 1), indicative of its role in transfer DNA synthesis [12,13]. Specifically, TraA_Ti_ of *R*. *bellii* has a MobA/L domain that probably has nickase activity similarly to the TrwC domain in *E*. *coli*, and the UrvD domain is shared by both *R*. *bellii* and *E*. *coli*. The main difference is that *R*. *bellii TraA*_*Ti*_ has a primase domain not present in TraI of *E*. *coli* (Fig 1). A possible role of primase in *R*. *bellii* TraA_Ti_ may be the initiation of DNA replication immediately following the generation of transfer ssDNA in the donor, or initiation of replication of the transfer DNA in the recipient. Thus far, the primase domain is unique to the relaxases of rickettsiae and insight into the function of this domain may identify a different mechanism in conjugation.

The conditions that stimulated high transcriptional activity of *traA*_*Ti*_ using *metG* as the reference gene were met when *R*. *bellii* was growing in ISE6 at 34°C for 72 hours (Fig 4), near the beginning of the stationary phase (Fig 1). In contrast, *R*. *bellii* did not show any changes in *traA*_*Ti*_ transcriptional activity when grown in mammalian cells. Possibly, cellular and metabolic processes of mammalian cells inhibited or indirectly altered the transcription of *traA*_*Ti*_. In nature, *R*. *bellii* has been observed to primarily infect arthropods, particularly ticks, so the increase in relative transcription found when grown in a tick cell line in the early stationary phase (Fig 2, 72 HPI) suggested the importance of the tick host in possible bacterial conjugation. Timing of transcription during the early stationary phase may prepare the bacteria for conjugal activity during this phase. This is in contrast to *E*. *coli* in which mating efficiency is maximal during rapid replication (mid-log phase) when *tra* transcripts are still detected although Tra proteins that are components of the transfer structure remain stable into stationary phase when TraM and TraI are lost [18].

The transcription of the other annotated *tra* genes analyzed was positively correlated with *traA*_*Ti*_. Although *traD*_*F*_ showed lower transcription activity overall, it still correlated positively with the other *tra* genes (Table 3), while transcription of RBE_0422 showed no correlation with the other *tra* genes despite the prediction that RBE_0422 and *traA*_*Ti*_ were part of an operon (S3 Table, [44–46]). All the *tra* genes were simultaneously up-regulated at early stationary phase or 72 HPI, further validating *metG* as the best reference gene (Table 3). *traF* mRNA was not detectable during the lag phase or at 12 HPI, but was present at the early stationary phase indicating that *traF* may not be important during the lag phase, but plays a role near the early stationary phase. Also, this shows that not all *tra* genes were transcribed together and may have different transcriptional regulation. A mutation in *traF* in *E*. *coli* eliminated formation of the pilus but did not affect replication, suggesting that pilus formation is not important for growth in *E*. *coli* [47], and this may also be true for *R*. *bellii traF*. *Rickettsia bellii traG* showed highest relative transcription near the early stationary phase indicating the importance of *traG* at this time and life stage. In *E*. *coli*, *traG* is multifunctional and participates in pilus assembly, mating pair stabilization, and entry exclusion, which further highlights this time point and stage of growth as a potential period to target for testing conjugation in *R*. *bellii* [11,48–50]. Taken together, our results suggest that testing for conjugation in *R*. *bellii* should be done in tick cell culture at later stages of infection.

Determining if the *tra* genes are functional as operons may be useful for understanding the regulation of these genes. There was evidence indicating that *traB*, -*V*, and -*C* may form an operon as suggested by overlapping of the start and stop codon of adjacent genes and similar degree of transcriptional up-regulation. Further evidence from the Database for prOkaryotic OperRons support *traB*, -*V*, and -*C* as an operon that also includes *traL* and-*E* (S3 Table). The Database for prOkaryotic OpeRons identified another *tra* operon that included *traU*, *trbC*, *traN*, -*F*, and -*H* (S3 Table) and genetic evidence show overlapping start and stop codons from *trbC*, *traN*, -*F*, and -*H* supporting these *tra* genes as belonging to an operon. The C_T_ values and fold increase of *traU*, *trbC*, *traN*, -*F*, and -*H* differed from each other despite evidence of an operon. However, differential and “segregational” decay of polycistronic transcripts are common and may explain the transcriptional pattern observed. The stability of transcripts can be affected by the secondary structure of RNA and/or the frequency of translation [51,52] highlighting the importance of proteomic analysis to support the regulation of transcription.

We considered RBE_0435 of *R*. *bellii* RML 369-C (S3 Fig) to be a possible candidate for the putative gene encoding the propilin (or pilin) subunits of the pilus. Searching for this protein homolog is difficult considering this coding gene is poorly conserved [53] although key characters such as a signal peptide and two transmembrane-spanning domain can help to identify potential homologs as seen with the homologous protein VirB2 [54]. Interestingly, the proteins involved in directing pilin to the periplasm (TraQ in *E*. *coli*) and the enzyme responsible for N-acetylation of pilin (TraX in *E*. *coli*) have not been found in *R*. *bellii* (S3 Table). This suggests that this potential pilin protein either does not require the modification and/or that it may not be involved in forming pili. Alternatively, the pili-like structures that have been observed in *R*. *bellii* [4] could originate from a different system, i.e. the VirB/D system. Contrarily, RBE_0435 locus has been illustrated to be the carboxyl-terminal end of *traK* [7]. The pili aspect of the *tra* system remains elusive.

If rickettsiae can engage in lateral gene transfer, then this could contribute new genes to the rickettsial genome, buffer the rate of reductive genome evolution [5] and enhance rickettsial fitness in new environments. The *tra* genes can facilitate import of new genes through recombination and insertion at a particular hot spot between *traA*_*Ti*_ and *traD*_*F*_, as suggested by Blanc et al. [10]. Evidence of this phenomenon is illustrated by the *tra* genes of *R*. *buchneri* where genes typical of Gram-positive aminoglycoside antibiotic biosynthesis gene clusters were found in a similar hot spot suggesting that the *tra* cluster can support piggybacking genes into the rickettsial genome to increase fitness and slow down reductive genome evolution [7]. Weinert *et al*. (2009) have pointed out that there is no evidence for the preferential transfer of genes between closely related species of rickettsiae and speculate that instead they may engage in “regenerative horizontal” DNA exchange further combatting a reductive genome evolution. This could be achieved by recombination of foreign DNA into the rickettsial genome, but recombination events have been argued to be generally rare in *Rickettsia* [55], although they seem more common in some species, e.g. *R*. *bellii* [56]. The possibility that the *tra* gene cluster can mediate acquisition of new genes and act as a hot spot for insertion to assist in the repair of damaged DNA in *R*. *bellii* through natural transformation is intriguing.

There is, however, ample evidence that some rickettsiae, especially those symbiotically associated with arthropods, harbor horizontally acquired genetic material on their plasmids that may have been acquired via bacterial conjugation. For example, *Rickettsia peacockii* maintains five genes in the pRPR plasmid that are distantly related to the *Pseudomonas aeruginosa* glycosylation island and may function in the uptake of dihydroxyacetone phosphate for phospholipid biosynthesis [57]. Furthermore, *R*. *buchneri* encodes a complete biotin operon on pREIS2 that shares homology with biotin operons recently acquired by horizontal gene transfer in *Neorickettsia risticii*, *N*. *sennetsu* and *Lawsonia intracellularis* [7]. Recently, an RTX type I secretion system (TISS) was found in *R*. *felis* plasmid pLSU-lb that was closely related to the TISS genes of *Cardinium* endosymbiont cBtQ1 of *Bemisia tabaci*, in which the RTX TISS genes were thought to originate through lateral gene transfer from *Vibrio* species [6]. In addition, other *R*. *bellii* isolates contain plasmids that have yet to be sequenced [1], and that may contribute to the dissemination of operons that mediate symbiosis. There are other mobile genetic elements such as the integrated genetic element found on the *R*. *buchneri* chromosome that was supported by bioinformatics to be closely related to and include the conjugation genes of *R*. *bellii* [7]. The acquisition of the *tra* genes may have been via a plasmid based on the recent discovery that *R*. *felis* LSU-lb harbors a plasmid containing the *tra* cluster closely related to pREIS3 of *R*. *buchneri*. The mechanism of transfer of an integrative conjugative element requires a functional integrase or recombinase that will excise the sequence from the chromosome followed by transfer strand synthesis and transport into the recipient either by encoding its own conjugation system or parasitizing another present in the donor [58,59]. Other strains of *R*. *bellii* have been documented to have a native plasmid [1] that could act as a vehicle for the spread of the *tra* genes, and it is conceivable that *R*. *bellii* RML 369-C may have lost its native plasmid through long term passage in cell culture [60]. Therefore, horizontally acquired functional genes and operons in rickettsiae may have been acquired in more than one way, and while having a native plasmid possibly increases the frequency at which genetic material is spread, it is likely not the only mechanism used. Thus far, the *tra* genes have not been demonstrated to function in bacterial conjugation and may be remnants of a once functional system; however, up-regulation of the *tra* genes in this research implies a potentially, functional conjugative and gene transfer system.

## Conclusion

We have demonstrated that the best reference gene out of the ten selected were *metG* or *metG* and *nrdF*. The same reference gene(s) can be used to standardize future relative transcriptional analysis of rickettsiae with similar treatments or to form a basis of potential reference genes with different treatments. Using the identified reference gene(s), we show that the transcription of the *tra* genes of *R*. *bellii* is highly upregulated at 72 HPI relative to 12 HPI only in tick cell culture, indicating that these genes are very active and contribute to the biology of *R*. *bellii*. Future research should explore the functions of the *tra* genes guided by bioinformatics to obtain detailed documentation of the mechanism of DNA mobility to and from the chromosome and plasmid, and the demonstration of the process of rickettsial conjugation.

## Supporting Information

S1 Fig Screenshots of Artemis of *Rickettsia rickettsii* Sheila Smith showing transcription levels of 5 genes: *metG*, *nrdF*, *gyrA*, *gltA*, and 16S rRNA. The order from left to right is of *metG* (A1G_05810), *nrdF* (A1G_03685), *gyrA* (A1G_01555), *gltA* (A1G_07170), and 16s rRNA gene (A1G_r07597). Each vertical black bar represents the hybridization level of mRNA from one probe on the array. The orientation and length of the genes are shown below the transcription peaks.
(TIF)

S2 Fig The relative transcription pattern of *metG* and *nrdF* to *traA*_*Ti*_. Relative transcription of *metG* and *nrdF* was analyzed using *traA*_*Ti*_ as a reference gene to show constitutive, but slightly varying transcription of both genes. Standard error bars are shown.
(TIF)

S3 Fig Amino acid alignment of TraA pilin. Sequences include TraA of *Escherichia coli* K-12 (CAA73225.1), TraA of *Enterobacter cloacae* (AKN35276.1), TraA of *Klebsiella pneumoniae* (CDO11547.1), TraA of *Salmonella enterica* subsp. enterica serovar Typhimurium (YP 009077414.1), RBE_0435 of *Rickettsia bellii* RML 369-C (WP 008579911.1), and RMA_0719 of *Rickettsia massiliae* MTU5 (ABV84869.1). Vertical lines indicate cleavage site of the signal peptide predicted by Phobius except for *R*. *bellii* and *R*. *massiliae*, which was predicted using Signal-BLAST. Underlined sequences show the transmembrane spanning membrane domain predicted using Phobius. “.” = same amino acid above it and “-” = space.
(TIF)

S1 Table Information about the genes investigated and the primer pairs. (XLSX)

S2 Table "Area under the curve" from RrSS tiling microarray for 5 genes selected. (XLSX)

S3 Table A comparison of predicted tra operons in *E*. *coli*, *R*. *bellii*, and *R*. *massiliae*. (XLSX)

S1 Text Supporting information methods. (DOCX)
