# Supplementary figures and images for: Transcriptional Analysis of the Conjugal Transfer Genes of Rickettsia bellii RML 369-C
Source: PLoS One. 2015 Sep 9;10(9):e0137214. doi: 10.1371/journal.pone.0137214 (PMC4564193; doi:10.1371/journal.pone.0137214)

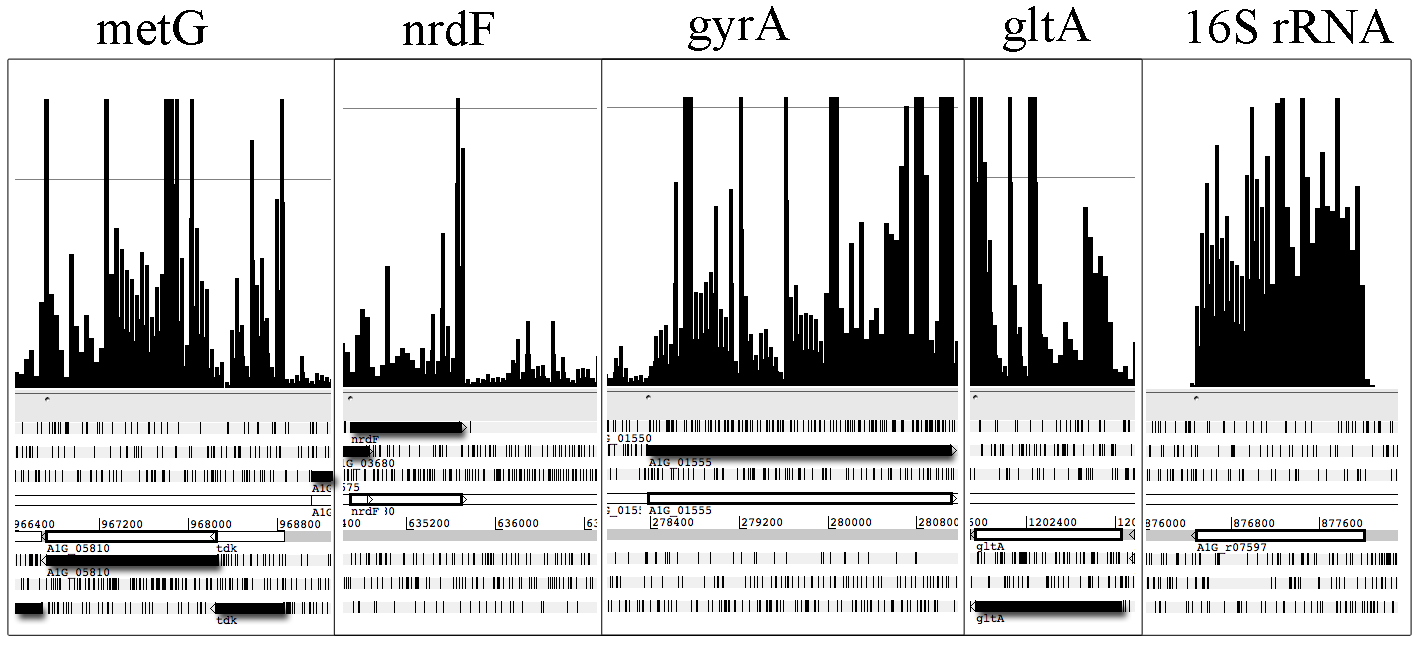

Supplement: S1 Fig — The order from left to right is of metG (A1G_05810), nrdF (A1G_03685), gyrA (A1G_01555), gltA (A1G_07170), and 16s rRNA gene (A1G_r07597). Each vertical black bar represents the hybridization level of mRNA from one probe on the array. The orientation and length of the genes are shown below the transcription peaks. (TIF) [file pone.0137214.s001.tif]

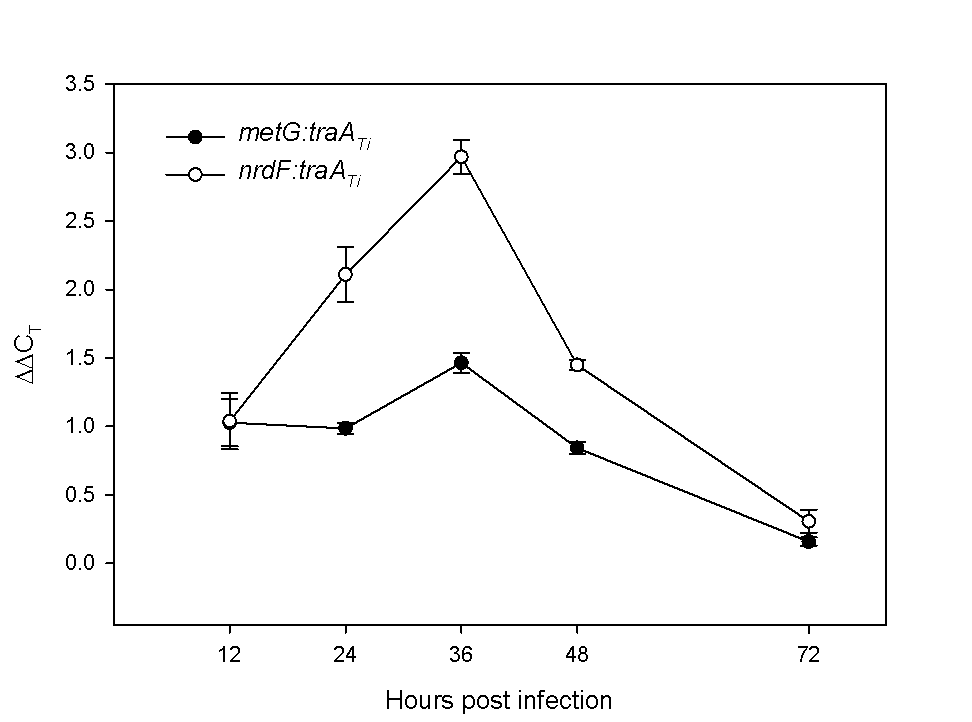

Supplement: S2 Fig — Relative transcription of metG and nrdF was analyzed using traA Ti as a reference gene to show constitutive, but slightly varying transcription of both genes. Standard error bars are shown. (TIF) [file pone.0137214.s002.tif]

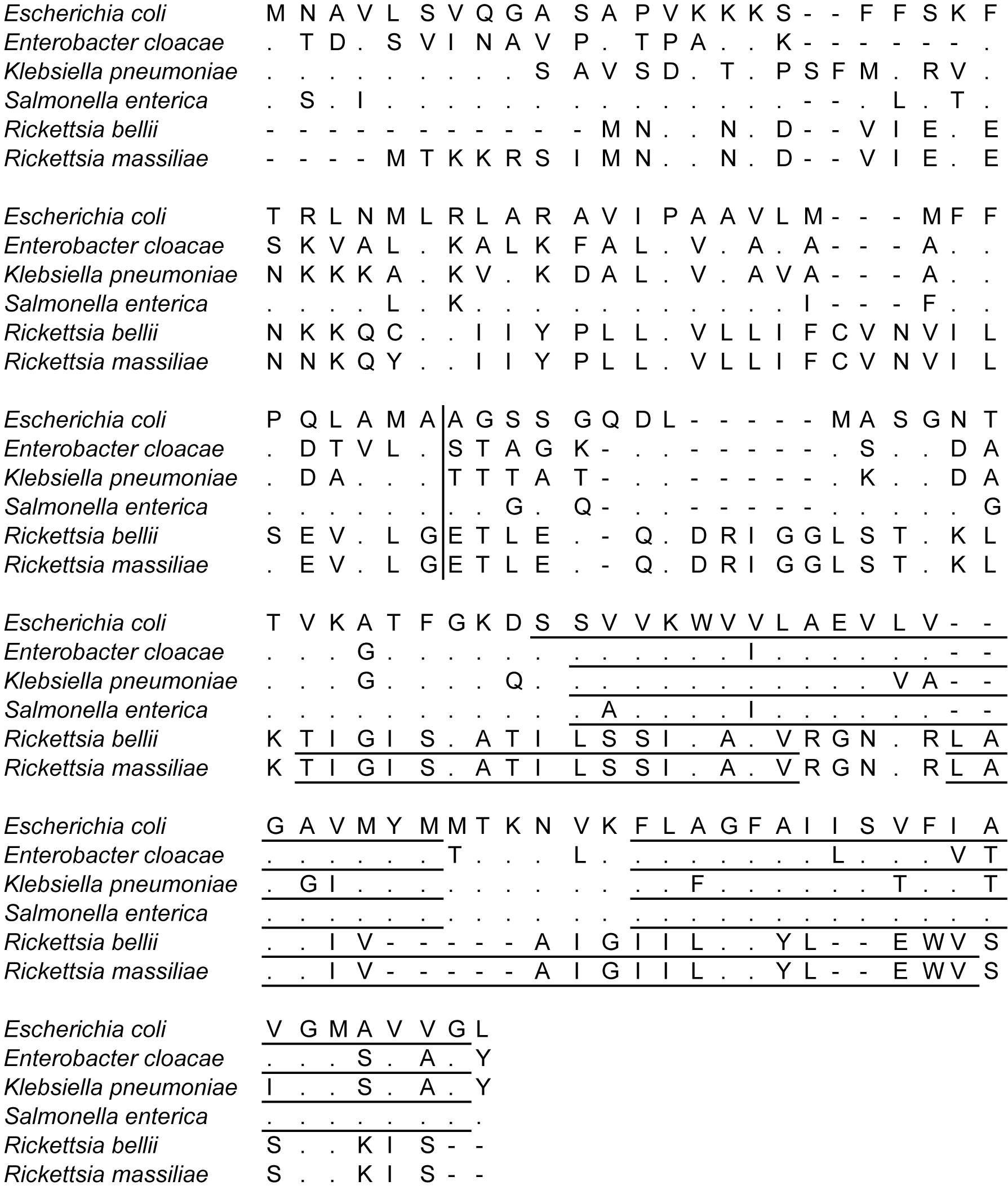

Supplement: S3 Fig — Sequences include TraA of Escherichia coli K-12 (CAA73225.1), TraA of Enterobacter cloacae (AKN35276.1), TraA of Klebsiella pneumoniae (CDO11547.1), TraA of Salmonella enterica subsp. enterica serovar Typhimurium (YP 009077414.1), RBE_0435 of Rickettsia bellii RML 369-C (WP 008579911.1), and RMA_0719 of Rickettsia massiliae MTU5 (ABV84869.1). Vertical lines indicate cleavage site of the signal peptide predicted by Phobius except for R. bellii and R. massiliae, which was predicted using Signal-BLAST. Underlined sequences show the transmembrane spanning membrane domain predicted using Phobius. “.” = same amino acid above it and “-” = space. (TIF) [file pone.0137214.s003.tif]
