# Supplementary material for: Transcriptional Analysis of the Conjugal Transfer Genes of Rickettsia bellii RML 369-C
Source: PLoS One. 2015 Sep 9;10(9):e0137214. doi: 10.1371/journal.pone.0137214 (PMC4564193; doi:10.1371/journal.pone.0137214)
Supplement: S1 Text — (DOCX) [file pone.0137214.s007.docx]

**S1 Text**

**Supporting information methods**

**Transcript levels of reference genes extrapolated from tiling microarray analysis of *Rickettsia rickettsii***

*R. bellii* reference genes (*metG*, *nrdF*, *gyrA*, *gltA*, and *16s rRNA* gene) were inferred to be transcribed above background by extrapolation from tiling array data of *Rickettsia rickettsii* strain Sheila Smith (RrSS) orthologous genes. Triplicate cultures of Vero cells in 75 cm^2^-flasks were grown to confluency at 37°C in RPMI1640 medium supplemented with 10% FBS, 25 mM HEPES buffer, and 0.25% NaHCO_3_, pH 7.5, inoculated with RrSS and incubated until cell layers were completely infected (3-4 days at the University of Minnesota Biosafety Level 3 Research Laboratory). RNA was then purified from these and from uninfected control cultures using Tri-Reagent (Sigma #T9424) according to the product protocol, briefly as follows: cells were dissolved in TriReagent; the aqueous phase was combined with an equal volume of isopropanol; the precipitated RNA was washed in 75% ethanol and then resuspended in water. RNA was directly labeled using the Kreatech ULS Labeling Kit (Leica Biosystem) according to the kit protocol. This method labels guanine nucleotides in RNA using platinum complexes linked with Cy3 fluorophores. Agilent (Santa Clara, CA) manufactured the whole genome tiling array for RrSS [GenBank CP000848.1] with designs we made using eArray, an array design tool provided by Agilent. Each slide consisted of eight arrays with 60,000 60mer oligonucleotides (probes), each with 15 nucleotides of overlap. The triplicate array data were quantile normalized and T-tested to determine significance (0.05). We used Artemis (http://www.sanger.ac.uk/) to help analyze the results of the arrays by superimposing the mean quantile normalized signal levels from each of the hybridized probes onto a graphical representation of the RrSS genome, such that transcription levels for each of the overlapping probes could be viewed in an entire genome context. The level of transcription is represented by the sum of the intensity of each probe within an annotated gene, using the calculated “area under the curve”. Numerical values of the “area under the curve” are displayed in Table S3.

**Relative transcription of *metG* and *nrdF* using *traA_Ti_* as a reference gene:**

The C_T_ values from *metG*, *nrdF*, and *traA_Ti_* from *R. bellii* grown in ISE6 were used **only** to demonstrate the constitutive transcription of *metG* and *nrdF* relative to *traA_Ti_* and **not** to imply that *traA_Ti_* is a good reference gene. The graph was created using SigmaPlot 9.0.

**Amino acid alignment of TraA (pilin):**

A search on the National Center for Biotechnology Information (NCBI) protein database using “TraA” and “pilin” resulted in 2945 total results. Out of the top 100, only one TraA sequence of a strain or species was selected as the representative of the different strains of the same species. *Escherichia coli* K-12 (CAA73225.1), TraA of *Enterobacter cloacae* (AKN35276.1), TraA of *Klebsiella pneumoniae* (CDO11547.1), TraA of *Salmonella enterica* subsp. enterica serovar Typhimurium, TraA *Providencia stuartii* (AFU34928.1*), Citrobacter freundii* (KJC03845.1), *Yersinia enterocolitica* LC20 (AHM76647.1), and *Citrobacter farmer* GTC 1319 (GAL51735.1) were chosen for further analysis with RBE_0435 of *R. bellii* RML 369-C and RMA_0719 of *R*. *massiliae* MTU5. Sequences were aligned using the default setting for MUSCLE alignment in MEGA 6 [1]. Signal peptide cleavage sites and transmembrane-spanning domains were predicted using Phobius [2] and Signal-BLAST [3]. The options selected for Signal-BLAST were “Gram-negative bacteria”, “SP1 - Best Sensitivity”, and “Detailed Analysis”. Subsequent analysis of cleavage sites and transmembrane-spanning domains supported excluding TraA sequences of *P. stuartii,*  *C. freundii,*  *C. farmer,* and *Y. enterocolitica* from the analysis.

References

1. Tamura K, Stecher G, Peterson D, Filipski A, Kumar S. MEGA6: Molecular Evolutionary Genetics Analysis version 6.0. Mol Biol Evol. 2013;30: 2725-2729. doi: 10.1093/molbev/mst197 [doi].

2. Kall L, Krogh A, Sonnhammer EL. A combined transmembrane topology and signal peptide prediction method. J Mol Biol. 2004;338: 1027-1036. doi: 10.1016/j.jmb.2004.03.016 [doi].

3. Frank K, Sippl MJ. High-performance signal peptide prediction based on sequence alignment techniques. Bioinformatics. 2008;24: 2172-2176. doi: 10.1093/bioinformatics/btn422 [doi].
